# Assessment of breast self- examination practice and associated factors among female workers in Debre Tabor Town public health facilities, North West Ethiopia, 2018: Cross- sectional study

**DOI:** 10.1371/journal.pone.0221356

**Published:** 2019-08-22

**Authors:** Asrat Hailu Dagne, Alemu Degu Ayele, Ephrem Mengesha Assefa

**Affiliations:** 1 Department of Midwifery, Debre Tabor University, Debre Tabor, Amhara Region, Ethiopia; 2 Department of Midwifery, Debre Tabor Health Sciences College, Debre Tabor, Amhara Region, Ethiopia; University of Buea, CAMEROON

## Abstract

**Background:**

Although breast Self-Examination is no longer tenable as a standard method to detect early breast cancer, world health organization recommends breast self -examination for raising awareness of women about breast cancer. Secondary prevention through monthly breast self-examination is the best option to tackle the rising incidence of breast cancer. Therefore, the aim of this study was to assess breast self -examination practice and associated factors.

**Methods:**

This cross-sectional study was conducted from April 23 to May 23, 2018. A total of 421 female workers in Debre Tabor Town public health facilities were included. The study participants were selected using simple random sampling technique from the study population. The collected data were checked for completeness. The data were entered and cleaned using EpiData version 3.1 then exported to SPSS version 20 for analysis. Crude odd ratio and probability value were identified for each independent variable and all independent variables with probability value of less than 0.2 were entered into multivariables logistic regression. Statistically significant associated factors were identified based on probability value (p-value) less than 0.05 and adjusted odd ratio with 95% confidence interval.

**Result:**

The mean age of participants was 25.2 (S.D = 4.12) and 137 (32.5%) of the participants had practiced breast self -examination and 64 (15.2%) of them performed it monthly. Family history of breast cancer (adjusted OR = 6.5, CI = 1.54–21.4), Knowledge about breast -self examination (adjusted OR = 5.74, CI = 2.3–14.4) and self- efficacy in practicing breast self -examination (adjusted OR = 4.7, CI = 1.84–12.11) were significantly associated with breast self -examination practice.

**Conclusions:**

The study showed that the prevalence of breast self-examination was low. Family history of breast cancer, knowledge about breast self -examination and self- efficacy in practicing breast self- examination did have statistically significant association with breast self—examination practice.

## Introduction

Breast self -examination is one of the early noticing way of breast cancer which involves the woman herself looking at and feeling each breast for possible mass, discharge, swelling, dimpling and other abnormalities [1]. Breast cancer is more common among women than men and it is a type of malignant tumor which begins in the cells of the breast [2].

Breast self -examination is a kind of examination made by each woman and it is cost effective, painless, easy to apply, safe, and non invasive procedures without special material or tool requirements. It is an important noticing way of breast cancer which takes five minutes to apply [3]. Breast cancer awareness improves the outcome of breast cancer treatment [4]

Breast self- examination is useful for women’s awareness of warning signs and symptoms of breast cancer like redness of the breast skin, changes in the size of the breast or nipple, a breast lump, pain in the breast or armpit, lump under the armpit, nipple rash, changes in the shape of the breast or nipple, bleeding or discharge from the nipple, pulling of the nipple, dimpling of the breast skin and changes in the position of the nipple [5].

Breast self -examination is also important to increase breast health awareness which helps to allow for timely detection of anomalies for those who do not have access to health facility and advanced laboratory investigations for diagnosing breast cancer [1].

It is known that clinical breast examination, Mammography, Ultrasound and Magnetic resonance imaging are breast cancer screening methods [6–11] and Mammography, Ultrasound and magnetic resonance imaging methods of breast cancer screening are not available in our study area. Using mammography instead of magnetic resonance imaging did not significantly increase screening sensitivity [8]. In clinical breast examination, the sensitivity, specificity and accuracy was 54%, 78% and 57% respectively [10]. The sensitivity of magnetic resonance imaging method of screening of breast cancer was higher than mammography and ultrasound [11].

Early identification of breast cancer through breast self -examination and diagnosis plays an important role in reducing its morbidity and mortality [9]. But most of women do not perform breast self- examination in low income countries because of lack of awareness and lack of knowledge of breast self- examination [12–16].

The incidence of breast cancer has been increasing in different regions of the world. The expected incidence rate of breast cancer is similar in different countries [17]. However, breast cancer mortality rate is much higher in low income countries than well developed countries due to lack of early screening methods and treatment of breast cancer [17, 18].

Worldwide, breast cancer is the second common cancer next to lung cancer and the fifth cause of cancer mortality [19, 20]. The incidence rate of breast cancer in North Africa and Sub-Saharan Africa (SSA) was 29.3 per 100000 and 22.4 per 100000 respectively. This incidence rate also indicated that breast cancer was increased between 2000 to 2015 [21]. According to breast cancer estimate in 2018, about 626,679 of women died from breast cancer in the world which accounted a crude mortality rate of 13 per 100.000 women [18].

About 10,000 Ethiopian women have already faced breast cancer and thousands of more patients are unreported as most women living in rural areas seek treatment from traditional healers before getting support from health facility [22].

Female health facility workers should be role model and they should educate the community to create awareness of early detection of signs and symptoms of breast cancer using breast self- examination. But health workers have not considered breast self- examination as a support for standard breast cancer screening method. Moreover, they had low practice of breast self examination [12, 23–26].

The findings of this study will help programme managers, stakeholders and health service providers to design appropriate intervention to increase practice of breast self- examination. Furthermore, it will also be helpful for better documentation of practice of breast self -examination of females working in public health facilities to design interventions aimed at reducing breast cancer mortality through increasing community awareness and improving early diagnosis and treatment of the disease.

Therefore, the aim of this study was to determine breast self- examination practice and identify associated factors among female workers in Debre Tabor Town public health facilities, North West Ethiopia.

## Methods

### Study design and setting

This cross- sectional study was conducted from April 23 to May 23, 2018 among females working in public health facilities of Debre Tabor Town. Debre Tabor is the capital of South Gondar Administrative Zone of Amhara Region, Ethiopia. There were 606 female workers in all health facilities. The town has one General hospital, three health centers and two colleges of health science. Female workers in Debre Tabor University Health Science College, Debre Tabor Health Science College of Amhara region, Debre Tabor General Hospital, Debre Tabor Health Center, Ginbot 20 Health Center and Hidar 11 Health Center were taken as study population.

### Sample size determination and sampling procedures

Sample size was calculated using single population proportion formula and the required sample size for this study was determined using the following assumptions; desired precision (d) = 5%, Confidence level = 95% (Zα/2 = 1.96 value) and 45.6% of the prevalence of breast self -examination practice among female health extension workers in Wolaita zone of Southern Ethiopia was taken [27]. Hence, the calculated sample size by considering 10% non- response rate was 421.

To collect the data, initially all female health facility workers were listed from each health facilities. Then, Simple random sampling technique was conducted using lottery method based on the proportion of the number of female workers in each health facility to select the samples.

### Data collection instrument and procedures

Data collection tool comprised of structured questionnaires that were prepared after thorough literature review and the local situation of the study area and purpose of the study were considered to prepare the questionnaire. Questionnaires were prepared first in English then translated to Amharic which is the vernacular language of the respondents by language expert for ease of understanding of the respondents. Data were collected via face- to- face interview technique using structured questionnaires.

Two nurses and two midwives who have BSc degree were selected and trained for data collection. They had previous exposure in data collection. Data were collected on socio-demographic, health and individual level related characteristics. Questionnaires were pretested on 22 (5%) of health facility workers of Nifas Mewucha Town in South Gondar Zone before final data were collected. The investigators and research assistants were involved to incorporate changes in questionnaires after pretest. To guarantee internal validity, only completed questionnaires were adopted.

### Measurement

Face- to- face interview questionnaires were used as data collection tool. The knowledge of the respondents was assessed by using 14 questions and the correct responses of each respondent for all questions were added to decide whether the respondent was knowledgeable or not.

The attitude, self- efficacy, barriers of breast self -examination and benefits of breast self -examination items were all answered as strongly agree, agree, neither agree nor disagree, disagree, and strongly disagree (on a five-point likert scale). Questionnaires related to attitude contain 6 questions with total score ranging from 6–30. Self- efficacy questionnaires include 10 questions with total score ranging from 10–50. Barriers of breast self- examination questionnaires also contain 8 questions with total score ranging from 8–40 and benefits of breast self -examination related questionnaires contain 4 questions with total score ranging from 4–20.

Therefore, the mean value of each variable for each respondent and the overall mean were identified to determine attitude, self- efficacy, and barriers of breast self -examination and benefits of breast self- examination.

### Data management

The collected data were checked for completeness. The data were entered and cleaned using EpiData version 3.1 then exported to SPSS version 20 for analysis. Descriptive analysis was employed to summarize the data. Crude odd ratio (OR) and probability value (P-value) were identified for each independent variable and all independent variables with probability value of less than 0.2 were entered into multivariables logistic regression. Statistically significant associated factors were identified based on probability value (p-value) less than 0.05 and adjusted odd ratio (AOR) with 95% confidence interval (CI).

### Operational definitions

**Knowledgeable**: participants who scored half and above values from all close-ended questions about the knowledge of breast self- examination [28].

**Positive attitude**: participants who scored mean and above values from all attitude-related questions towards breast self- examination [26].

**Self- efficacy in practicing breast self -examination**: participants who scored mean and above values from all close-ended questions of self- efficacy of breast self -examination [29].

**Not Knowledgeable**: participants who did not score half and above values from all close-ended questions about the knowledge of breast self- examination.

**Negative attitude**: participants who did not score mean and above values from all attitude-related questions towards breast self -examination.

**Participants who did not self -efficacy in practicing breast self- examination**: participants who did not score mean and above values from all close-ended questions of self- efficacy of breast self -examination.

**Barrier of breast self -examination**: participants who scored mean and above values from all close-ended questions of the barriers of breast self- examination [29].

**Benefits of breast self -examination**: participants who scored mean and above values from the total of benefits of breast self -examination related questions [29].

**Breast self- examination Performed** means breast self- examination was performed monthly during menses (regular) or ever performed breast self -examination (irregular) [29].

### Ethics approval and consent to participate

Ethical clearance was obtained from Institutional Review Board of Debre Tabor University, health Science College. Formal letter of cooperation was written for Debre Tabor town health facilities. We stated for the participants that they had the right of unwilling to participate in the study and they had also the right to quit their participation at any stage without any restriction. Moreover, we informed the purpose, procedures, advantage and disadvantage of the study to the participants. Finally, informed written consent was obtained from each study participants.

## Result

### Socio-demographic characteristics

In this study, there were a total of 421 respondents with a response rate of 100%. The respondents’ age ranges from 20–50 years. The mean age of the study population was 25.3 (SD ± 4.12) years. Majority of respondents were Orthodox (87.4%) and 53 (12.6%) of participants were Muslim. Among participants, 252 (60%) were unmarried and 169 (40%) were married (see Table 1).

**Table 1:** Socio-demographic characteristics of female workers in Debre Tabor town’s public health facilities, South Gondar Zone, 2018.

Variables	Frequency (n = 421)	Percent (%)
**Marital status**		
Married	**252**	**60**
Single	169	40
**Maternal age**		
20–30	193	45.8
31–40	88	21
41–50	140	33.2
**Woman’s educational status**		
Less than12^th^ grade	67	16
Grade 12 complete and diploma 12 grade and Diploma	253	60
BSc and above	101	24
**Husband’s Occupation**		
Government Employee	127	50.4
Non-governmental employee	59	23.4
Trader	66	26.2
**Religion**		
Orthodox	369	87.6
Muslim	52	12.4
**Ethnicity**		
Amhara	397	94.3
Others	24	5.7
**Income per month**		
Less than 22 US dollar	29	6.9
22.04–59	85	20.2
59.1–187.5	202	48
Greater than 187.5	105	24.9
**Year of experience**		
1–5 years	225	53.4
6–15 years	117	27.8
More than 15 years	79	18.8
**Profession**		
Non- health professionals	158	37.53
Nurse	169	40.14
Others	94	22.33

Others (ethnicity) = Oromo, Tigre and Kimant; Others (profession) = Doctors, midwives, laboratory technicians and technologists, Pharmacy technician and Pharmacists and anesthetist

### Practice, knowledge and attitude on breast self-examination

From the total sample of 421, 32.5% of respondents had ever practiced breast self- examination and only 64 (15.2%) of them practiced breast self-examination regularly or monthly. The other 73 (17.3%) of participants had practiced breast self- examination irregularly. Among the total respondents, 167 (39.7%) of them had self- efficacy in practicing breast self- examination. Majority of the participants (70.3%) scored more than half of the knowledge question and 77.4% of the respondents had positive attitude (see Table 2).

**Table 2:** Practice, knowledge, attitude and health related characteristics towards breast self-examination among female workers in Debre Tabor Town public health facilities, South Gondar Zone, 2018.

Variables	Frequency (n = 421)	Percent (%)
**Practice breast self- examination**		
Regular	64	15.2
Irregular	73	17.3
Never practice breast self- examination	284	67.5
**Knowledge**		
Knowledgeable	296	70.3
Not knowledgeable	125	29.7
**Attitude**		
Have positive attitude	326	77.4
Have negative attitude	95	12.6
**Family history of breast cancer**		
Yes	23	5.4%
No	398	94.8
**Knowing someone with breast cancer**		
Yes	266	63.2
No	155	36.8
**Use of contraceptive method**		
Users of hormonal contraceptives	351	83.4
Users of barrier methods and others	46	10.9
Non users of contraceptive methods	24	5.7
**Barriers of breast self examination**		
Yes	239	56.8
No	182	43.2
**Regular menstrual period**		
Yes	327	77.7
No	94	22.3
**Self- efficacy in PBSE**		
Yes	167	39.7
No	254	60.3
**Identify benefits of BSE**		
Yes	305	72.4
No	116	27.6

Others (use of contraceptive method) = Lactation amenorrhea method, Rhythm method, tubal ligation and vasectomy; PBSE = Practicing breast self- examination and BSE = Breast self -examination

### Factors associated with breast self -examination practice

Factors found to be associated with breast self- examination practice using bivariate analysis were marital status, husband’s occupation, age of female workers, family income, year of experience, profession, knowledge of the respondents, attitude of the respondents, family history of breast cancer, personal history of breast cancer, barriers of breast self- examination, self- efficacy in practicing breast self- examination and benefits of breast self- examination.

Moreover, we found that knowledge of female health facility workers towards breast self- examination, family history of breast cancer and self- efficacy in practicing breast self- examination had statistically significant association with breast self- examination practice.

The odds of practicing breast self- examination was 5.74 (AOR, 95% CI: 2.3–14.4) times higher among knowledgeable female workers for breast self- examination compared to female workers who were not knowledgeable. Female health facility workers who had family history of breast cancer and self- efficacy in practicing breast self- examination were 6.5 (AOR, 95% CI: 1.54–21.4) and 4.7 (AOR, 95% CI: 1.84–12.11) times more likely to practice breast self- examination compared to those female health facility workers who had no family history of breast cancer and self- efficacy in practicing breast self- examination respectively (see Table 3).

**Table 3:** Bivariate and multivariables analysis of associated factors of breast self- examination of female workers in Debre Tabor Town public health facilities, South Gondar Zone, 2018.

Variables	Perform BSE		Crude OR (95%CI)	Adjusted OR (95%CI)
	Yes	No		
**Profession**				
Not health professional	38	120	1	
Nurse	70	99	***2.23(1.1–4.7)	2.4 (0.89–9.7)
Others	29	65	**1.40(0.9–4. 33)	1.6 (0.67–4.8)
**Knowledge**				
Knowledgeable	115	181	***2.97(1.7–10.18)	***5.74 (2.3–14.4)
Not knowledgeable	22	103	1	
**Attitude**				
Positive attitude	114	212	**1.68(1.32–6.5)	**3.1 (0.76–2.38)**
Negative attitude	23	72	1	
**Family history of breast cancer**				
Yes	19	4	***11.27 (6.12–17.29)	**6.5 (1.54–21.4)
No	118	280	1	
**Self- efficacy in PBSE**				
Yes	110	57	***16.2(8.7–27.3)	**4.7 (1.84–12.11)
No	27	227	1	

**P-Value≤0.01 and

***P-Value≤0.001

Others (Profession) = Doctors, midwives, laboratory technicians and technologists, Pharmacy technicians and pharmacists, anesthetists; PBSE = Practicing breast self- examination and BSE = Breast self- examination

## Discussion

According to the result of this study, 32.5% of participants had ever practiced breast self- examination and this practice was generally low. This might be due to health professionals concerned that breast self- examination is not a standard method to detect breast cancer but they should consider breast self- examination for the early awareness of signs and symptoms of breast cancer. A similar finding was found in the studies which were conducted in Iran and Nigeria [14, 29]. This could be because of knowledge or awareness of study participants and similarity of study settings. But the finding of this study was lower than the studies conducted in Malaysia, Turkey, Cameroon, Nigeria and among female health extension workers in wolaita zone, Southern Ethiopia [27, 30–33]. The reason for this might be due to the difference in considering breast self- examination for the awareness of signs and symptoms of breast cancer, knowledge difference of study participants and the difference between study areas.

The odds of breast self- examination practice among women who were knowledgeable were 5.74 times higher than women who were not knowledgeable. A similar result was found in a study conducted in Malaysia and among female health professionals in Western Ethiopia [28, 30]. This is due to knowledgeable respondents’ motivation to practice breast self-examination.

The study indicated that the odds of breast self- examination among women who had family history of breast cancer were 6.5 times higher among women who did not have family history of breast cancer. This finding is in line with the study conducted in Ethiopia [28]. This could be due to getting information, their better awareness towards severity of the disease and advice on breast self- examination in noticing any change at early stage. Therefore, awareness of women towards breast self- examination increases if they had family history or personal history of breast cancer.

This study also showed that the odds of breast self- examination among female health facility workers who had self- efficacy in practicing breast self- examination were 4.7 times better than female health facility workers who did not have self- efficacy in practicing breast self- examination. The finding of this study is consistent with the study conducted in Iran [29, 31]. This is due to respondents who had self- efficacy had knowledge and skill to perform breast self- examination.

### Limitation

There could be possibility of recall bias because of the participants were not actually assessed on their ability to correctly perform breast self-examination.

## Conclusions

The study showed that the prevalence of breast self-examination was low. Family history of breast cancer, knowledge about breast- self examination and self- efficacy in practicing breast self- examination do have statistically significant association with breast self- examination practice. Therefore, there is a need for health intervention on breast self- examination and self- efficacy to increase breast self- examination practice for the awareness of women towards breast cancer.

## Supporting information

S1 Tools The questions or questionnaire used in the study were both Amharic and English language. (DOCX)
