# Supplementary material for: Assessment of breast self- examination practice and associated factors among female workers in Debre Tabor Town public health facilities, North West Ethiopia, 2018: Cross- sectional study
Source: PLoS One. 2019 Aug 22;14(8):e0221356. doi: 10.1371/journal.pone.0221356 (PMC6705765; doi:10.1371/journal.pone.0221356)
Supplement: S1 Tools — (DOCX) [file pone.0221356.s001.docx]

**COMPLETE CONSENT FORM AND**

**Questionnaires for the study**

The researcher explained the aim of the study. Moreover, I can decide any time if I do not want to participate. Therefore, I assure that my interest to participate in this study is truly from my knowledge.

If client refuses, please check using X mark in this box

Signature of Person administering consent_______________

Date______________________

Client’s Signature (If agree to be involved)_____________

Date_____________________

Identification number: ______________________________

Data collector: _____________________

| **Section 1:Sociodemographic characteristics of female health facility workers** | | | | | | | | | | |
| --- | --- | --- | --- | --- | --- | --- | --- | --- | --- | --- |
| 101 | | Age | | | | ________________year | | | | |
| 102 | | Marital status | | | | 1. Single 2. Married 3. Divorced 4. Widowed 5. Separated 6. Cohabited | | | | |
| 103 | | Number of pregnancy | | | | ------------------------ | | | | |
| 104 | | Education status of husband | | | | - - - 1. No formal education       2. Primary school completed       3. Secondary school completed       4. College or university completed | | | | |
| 105 | | occupational status of husband | | | | 1. Farmer 2. House wife  3.Government employ 4.Student  5.Trader 6.Daily labored  7.No job 8.Specify if other------------------------------------------------------------------------------ | | | | |
| 106 | | Religion | | | | 1.Orthodox  2.Muslim  3.Protestant  4.Catholic  5. Specify if other----------------------------------- | | | | |
| 107 | | Ethnicity | | | | 1.Amhara  2.Tigre  3.Oromo  4.Specify if other---------------------------------------- | | | | |
| 108 | | Level of education of female health facility worker | | | | 1.<12 grade  2. 12 grade and diploma  3. BSc and above | | | | |
| 109 | | What is your monthly income? | | | | --------------------------Ethiopian Birr per month | | | | |
| 110 | | What is your source of income?  (more than one answer is possible) | | | | 1. monthly salary 2. private business ( drug shope, clinic, hospital) 3. part time working 4. Specify if other------------------------------------------------------ | | | | |
| 111 | | Year of experience | | | | -------------------------------year | | | | |
| 112 | | What is your profession | | | | 1. Not health professional  2. Nurse  3. Midwife  4. Doctor  5. Laboratory technicians or technologists  6. Pharmacy technicians or pharmacists  7. Anesthetists  8.Specify if other------------------------------------ | | | | |
| **Section 2:Health related factors** | | | | | | | | | | |
| 201 | | How often do you practice breast self examination? | | | 1.regularly (monthly during menses)  2. Irregularly ( not monthly and not during menses)  3.I never practice breast self examination | | | | | |
| 202 | | Do you have family history of breast cancer? | | | 1.Yes  2.No | | | | | |
| 203 | | Do you have family history of cancer? | | | 1.yes  2.No | | | | | |
| 204 | | Do you have personal history of breast cancer? | | | 1.Yes  2.No | | | | | |
| 205 | | Which contraceptive method do you use? | | | 1. Oral contraceptive  2. Injectables contraceptive  3. Implants contraceptive  4.Condom  5.IUCD  6.Lactational amenorrhea methods  7.Rythum methods  8.Tubal ligation and vasectomy  9. Specify if other------------------------------------------------- | | | | | |
| 206 | | Is your menstrual period regular? | | | 1.Yes  2.No | | | | | |
| **Section 3:Individual level related factors questionnaires of breast self examination practice among female health facility workers in South Gondar Zone, North West Ethiopia**  **3.1.Knowledge related questionnaires** | | | | | | | | | | |
| 301 | | | | Which sex group does breast cancer affect? | | | | 1.Female only  2.Male only  3.Both male and female are affected | | |
| 302 | | | | Does early detection improve chance of survival? | | | | 1. Yes 2. No | | |
| 303 | | | | Is breast Cancer curable if detected at early stage? | | | | 1.Yes  2. No | | |
| 304 | | | | Do you Know types of breast cancer screening methods? | | | | 1.Yes  2. No | | |
| 305 | | | | Which types of breast cancer screening methods do you know?  (More than one answer is possible) | | | | Breast self examination  Clinical breast examination  Mammography  Ultrasound  Magnetic resonance imaging | | |
| 306 | | | | Have you ever heard breast self examination? | | | | Yes  No | | |
| 307 | | | | Who perform breast self examination? | | | | Self  Health professionals  Relatives | | |
| 308 | | | | When should a girl begin breast self examination? | | | | 1.Less than 20 years  2.Above 20 years  3. 20-30 years  5.Specify if others----------------------------------- | | |
| 309 | | | | How often breast self examination performed?? | | | | 1.Weekly  2.Monthly  3.Yearly  4.I do not know | | |
| 310 | | | | What do you look during breast self examination? (More than one answer is possible) | | | | 1. Breast lamp 2. Size of the breast 3. Change in nipple and unusual discharge 4. Change in skin colour | | |
| 311 | | | | What is the examination technique applied during breast self examination? | | | | 1. Inspection 2. Palpation 3. Inspection and palpation 4. I do not know | | |
| 312 | | | | How is breast self-examination done? | | | | 1. With palm and three middle fingers 2. Palpate with any of the fingers 3. I don’t know | | |
| 313 | | | | What is the advantage of regular breast self-examination? | | | | 1. Detect any abnormality 2. Learn how the breast normally looks and feels 3. Detect breast cancer earlier and promote treatment | | |
| 314 | | | | Do you ever taught breast self-examination to a client or relatives? | | | | 1. Yes 2. No | | |
| **3.2.Attitude related questionnaires** | | | | | | | | | | |
| 315 | | | Breast cancer is highly prevalent and it is a leading cause of deaths among all cancers in Ethiopia. | | | | | 1.Strongly disagree  2.Disagree  3.Niether agree nor disagree  4.Agree  5.Strongly agree | | |
| 316 | | | Any woman including  you can acquire breast cancer | | | | | 1.Strongly disagree  2.Disagree  3.Niether agree nor disagree  4.Agree  5.Strongly agree | | |
| 317 | | | Breast cancer cannot  be transmitted | | | | | 1.Strongly disagree  2.Disagree  3.Niether agree nor disagree  4.Agree  5.Strongly agree | | |
| 318 | | | Breast self examination helps in prevention of breast cancer | | | | | 1.Strongly disagree  2.Disagree  3.Niether agree nor disagree  4.Agree  5.Strongly agree | | |
| 319 | | | Breast self examination causes no harm | | | | | 1.Strongly disagree  2.Disagree  3.Niether agree nor disagree  4.Agree  5.Strongly agree | | |
| 320 | | | Doing breast self examination is not unpleasant | | | | | 1.Strongly disagree  2.Disagree  3.Niether agree nor disagree  4.Agree  5.Strongly agree | | |
| **3.3.Self efficacy related questionnaires** | | | | | | | | | | |
| 321 | You know how to perform breast self examination. | | | | | | 1.Strongly disagree  2.Disagree  3.Niether agree nor disagree  4.Agree  5.Strongly agree | | | |
| 322 | You can perform breast self examination correctly | | | | | | 1.Strongly disagree  2.Disagree  3.Niether agree nor disagree  4.Agree  5.Strongly agree | | | |
| 323 | You could find a breast lump by performing breast self examination | | | | | | 1.Strongly disagree  2.Disagree  3.Niether agree nor disagree  4.Agree  5.Strongly agree | | | |
| 324 | You are able to find a breast lump that is the size of a walnut | | | | | | 1.Strongly disagree  2.Disagree  3.Niether agree nor disagree  4.Agree  5.Strongly agree | | | |
| 325 | You are able to find a breast lump that is the size of a hazelnut | | | | | | 1.Strongly disagree  2.Disagree  3.Niether agree nor disagree  4.Agree  5.Strongly agree | | | |
| 326 | You are able to find a breast lump that is the size of a pea | | | | | | 1.Strongly disagree  2.Disagree  3.Niether agree nor disagree  4.Agree  5.Strongly agree | | | |
| 327 | You are sure of the steps to follow for doing breast self examination | | | | | | 1.Strongly disagree  2.Disagree  3.Niether agree nor disagree  4.Agree  5.Strongly agree | | | |
| 328 | You are able to tell something is wrong with my breast when doing breast self examination | | | | | | 1.Strongly disagree  2.Disagree  3.Niether agree nor disagree  4.Agree  5.Strongly agree | | | |
| 329 | You are able to tell something is wrong with my breast when I look in the mirror | | | | | | 1.Strongly disagree  2.Disagree  3.Niether agree nor disagree  4.Agree  5.Strongly agree | | | |
| 330 | You can use the correct part of your fingers when examining your breast | | | | | | 1.Strongly disagree  2.Disagree  3.Niether agree nor disagree  4.Agree  5.Strongly agree | | | |
| **3.4.Barriers of breast self examination related questionnaires** | | | | | | | | | | |
| 331 | Breast self examination is embarrassing to me | | | | | | | | 1.Strongly disagree  2.Disagree  3.Niether agree nor disagree  4.Agree  5.Strongly agree | |
| 332 | Beast self examination takes too much time | | | | | | | | 1.Strongly disagree  2.Disagree  3.Niether agree nor disagree  4.Agree  5.Strongly agree | |
| 333 | It is hard to remember to do breast examination | | | | | | | | 1.Strongly disagree  2.Disagree  3.Niether agree nor disagree  4.Agree  5.Strongly agree | |
| 334 | I don't have enough privacy to do breast examination | | | | | | | | 1.Strongly disagree  2.Disagree  3.Niether agree nor disagree  4.Agree  5.Strongly agree | |
| 335 | Breast self examination is not necessary if you have a breast exam by a healthcare provider | | | | | | | | 1.Strongly disagree  2.Disagree  3.Niether agree nor disagree  4.Agree  5.Strongly agree | |
| 336 | Breast self examination is not necessary if you have a routine mammogram | | | | | | | | 1.Strongly disagree  2.Disagree  3.Niether agree nor disagree  4.Agree  5.Strongly agree | |
| 337 | My breast is too large for me to complete breast self examination | | | | | | | | 1.Strongly disagree  2.Disagree  3.Niether agree nor disagree  4.Agree  5.Strongly agree | |
| 338 | I have other problems more important than doing breast self examination | | | | | | | | 1.Strongly disagree  2.Disagree  3.Niether agree nor disagree  4.Agree  5.Strongly agree | |
| **Section 3.5:Benefits of breast self examination related questionnaires** | | | | | | | | | | |
| 339 | When I do breast self examination, I am doing something to take care of myself | | | | | | | | | 1.Strongly disagree  2.Disagree  3.Niether agree nor disagree  4.Agree  5.Strongly agree |
| 340 | Completing breast self examination each month may help me find breast lumps early | | | | | | | | | 1.Strongly disagree  2.Disagree  3.Niether agree nor disagree  4.Agree  5.Strongly agree |
| 341 | Regular breast self examination decreases the rate of death from breast cancer | | | | | | | | | 1.Strongly disagree  2.Disagree  3.Niether agree nor disagree  4.Agree  5.Strongly agree |
| 342 | If I find a lump early through breast self examination, my treatment for breast cancer may not be as bad | | | | | | | | | 1.Strongly disagree  2.Disagree  3.Niether agree nor disagree  4.Agree  5.Strongly agree |

እኔ ከዚህ በታች በፊርማዬ ያረጋገጥኩት በዚህ ጥናት ተሳታፊ ለመሆን ስወስን የጥናቱ አላማዎች፤አሰራር እና ቅድመ ሁኔታዎች በግልጽ በመረዳት እና እንዲሁም ከጥናቱ ተሳታፊነት ፈቃደኝነቴን በማንኛውም ደረጃ የማቋረጥ መብቴን በማረጋገጥ ነው፡፡ በጥናቱ ተሳታፊ መሆኔን በፊርማዬ እያረጋገጥኩ እነዚህ መረጃዎች ሁሉ በሚገባ በምረዳው ቋንቋ የተገለጸልኝ መሆኑን በፊርማዬ አረጋግጣለሁ፡፡

የጥናቱ ተሳታፊ ፊርማ------------------------------------------------

ቀን----------/----------/2010 ዓ.ም

የመረጃ ሰብሳቢ ስም-----------------------------------------

መረጃውን የሰበሰበው ሰው ፊርማ--------------------------------------------------------------------

መለያ ቁጥር-------------------------------------------------------------------

**ከዚህ በታች በሰንጠረዥ ውስጥ ላሉት ጥያቄዎች በተሰጠው ባዶ ቦታ ላይ በመጻፍ ለጥያቄው መልስ ይስጡ ወይም ከተሰጡት ምርጫ ላላቸው ጥያቄዎች ደግሞ መልሳቸው ላይ ሰርክል (ክብ) ምልክት ያድርጉ፡፡**

| **ክፍል 1: ማህበረሰባዊ እና ግላዊ የህይወት ታሪክን የያዘ ቃለ-መጠይቅ ነዉ** | | | | | |
| --- | --- | --- | --- | --- | --- |
| 101 | | እድሜ | | | ________________አመት |
| 102 | | የጋብቻ ሁኔታ | | | 1. ያገባ/ች 2. ያላገባ/ች 3. የተፋታ/ች 4. ባል / ሚስት የሞተበት 5. ተለያይተው የሚኖሩ 6. ሳይጋቡ አብረው የሚኖሩ |
| 103 | | ስንተ ልጆች አሎት? | | | ----------------------------- |
| 104 | | የባል ትምህርት ደረጃ | | | 1 ያልተማረ/ች  2. አንደኛ ደረጃ ትምህርት ያጠናቀቀ/ች  3. ሁለተኛ ደረጃ ትምህርት ያጠናቀቀ/ች  4. ኮሌጅ እና ከዚያ በላይ ያጠናቀቀ/ች |
| 105 | | የባል ስራ ሁኔታ | | | 1. የመንግስት ሰራተኛ 2. የቤት እመቤት  3. ነጋዴ 4.ተማሪ  5.ስራ የሌለው 6.የቀን ሰራተኛ  7. አርሶ አደር 8. ሌላ ከሆነ ይግለጹ------------------------------------------------------------------------------ |
| 106 | | ሃይማኖት | | | 1.ኦርቶዶክስ  2.ሙስሊም  3.ፕሮቴስታንት  4.ካቶሊክ  5. ሌላ ከሆነ ይግለጹ----------------------------------- |
| 107 | | ብሄረሰብ | | | 1.አማራ  2.ትግሬ  3.ኦሮሞ  4. ሌላ ከሆነ ይግለጹ--------------------------------------- |
| 108 | | የእርሶ ትምህርት ደረጃ | | | 1.ከ 12ኛ ክፍል በታች  2. 12ኛ ክፍልና ዲፕሎማ  3. ዲግሪና ከዚያ በላይ |
| 109 | | የወር ገቢዎ ምን ያህል ነው? | | | -------------------------የኢትዮዽያ ብር በወር |
| 110 | | የገቢዎ ምንጭ ምንድነው?  (ከአንድ በላይ መልስ ሊኖረው ይችላል) | | | 1. የወር ደሞዝ 2. የግል ስራ ( ሱቅ፤, ክሊኒክ, ሆስፒታል ወዘተ…) 3. የቀን ስራ 4. ሌላ ከሆነ ይግለጹ------------------------------------------------------ |
| 111 | | የስራ ልምድ | | | -------------------------------አመት |
| 112 | | የሙያ ዘርፍ | | | 1.የጤና ባለሙያ አይደለሁም  2.ነርስ  3. ሚድዋይፍ  4. ዶክተር  5.ላብራቶሪ ቴክኒሽያን ወይም ቴክኖሎጂስት  6. ፋርማሲ ቴክኒሽያን ወይም ፋርማሲስት  7.ሰመመን ሙያተኛ ወይም አንስቴሲስት  8. ሌላ ከሆነ ይግለጹ----------------------------------- |
| **ክፍል 2:ከጤና ጋር የተያያዙ ጥያቄዎችን በተመለከተ** | | | | | |
| 201 | በምን ያህል ጊዜ የራሶን ጡት በራሶ ምርመራ ያደርጋሉ? | | | | 1. በቁዋሚነት (በየወሩ የወር አበባ ሲኖረኝ)  2. በማንኛውም ጊዜ (ቁዋሚ የመመርመሪያ ጊዜ የለኝም))  3. በፈጹም ለራሴ ምርመራ አድርጌ አላውቅም |
| 202 | በእርሶ ቤተሰብ በጡት ካንሰር የተያዘ ሰው ያውቃሉ? | | | | 1.አዎ  2.የለም |
| 203 | በእርሶ ቤተሰብ በካንሰር የተያዘ ሰው ያውቃሉ? | | | | 1.አዎ  2.የለም |
| 204 | እርሶ በጡት ካንሰር ተጠቅተው ያውቃሉ? | | | | 1.አዎ  2.የለም |
| 205 | እርሶ የትኛውን የርግዝና መከላከያ ዘዴ ነው የሚጠቀሙት? | | | | 1.በአፍ የሚዋጥ ክኒን  2.በመርፌ የሚወሰድ  3. በክንድ የሚደረግ  4. ኮንዶም  5. በማህጸን የሚደረግ  6. ጡት በማጥባት  7. የወር አብባን በቁዋሚነት በመቆጣጠር  8. የማህጸንና የወንድ የዘር ፍሬን በዘላቂነት መቁዋጠር  9.ሌላ ከሆነ ይግለጹ--------------- |
| 206 | የወር አበባዎ በየወሩ ይመጣል? | | | | 1.አዎ  2.የለም |
| **ክፍል 3:በግል ደረጃ የተመለከቱ ጥያቄዎች**  **3.1.-የሰራተኞችን ግንዛቤ (እውቀት)ን የሚመለከቱ ጥያቄዎች** | | | | | |
| 301 | በጡት ካንሰር የሚያጠቃው ጾታ የትኛው ነው? | | | 1.ሴት ብቻ  2. ወንድ ብቻ  3. የጡት ካንሰር ወንድና ሴትን ያጠቃል | |
| 302 | የጡት ካንሰር በቶሎ መታወቁ በህይወት የመኖር ጊዜን ያሻሽላል? | | | 1. አዎ 2. የለም | |
| 303 | የጡት ካንሰር በቶሎ በመታወቁ መዳን ይቻላልን? | | | 1. አዎ 2. የለም | |
| 304 | የጡት ካንሰርን የመለያ ዘዴዎችን ያውቃሉ? | | | 1. አዎ 2. የለም | |
| 305 | ለ 304ኛ ጥያቄ መልሶ አዎን ከሆነ የትኛውን የጡት ካንሰር መለያ ያውቃሉ? (ከአንድ በላይ መልስ መመለስ ይቻላል) | | | የጡት ካንሰርን በራስ ለይቶ ማወቅ  በክሊኒካል ጡትን መመርመር  ማሞግራፊ  አልትራሳዎንድ  ማግኔቲክ ሬዞናንት ኢሜጂንግ | |
| 306 | የጡት ካንሰርን በራስ ለይቶ ማወቅ የሚለውን ከዚህ በፊት ሰምተው ያውቃሉ? | | | 1. አዎ 2. የለም | |
| 307 | የጡት ካነሰርን በራስ ለይቶ የሚያውቀው ማነው?. | | | የራስን ጡት በመመርመር  የጤና ሙያተኛ  ጉዋደኛ | |
| 308 | አንዲት ልጃገረድ መቼ ነው የራሱዋን ጡት መመርመር የምትጀምረው ? | | | 1. ከ 20 አመት በታች  2. ከ 20 አመት በላይ  3 .ከ 20-30 አመት  4. ሌላ መልስ ካሎት ይግለጹ ---------------------------------- | |
| 309 | በየስንት ጊዜ ነው የጡት ካንሰር በራስ የሚመረመረው ? | | | 1.በየሳምንቱ  2.በየወሩ  3. በየአመቱ  4. አላውቅም | |
| 310 | የራስዎን ጡት ሲመረምሩ የሚመለከቱት ነገር ምንድነው? (ከአንድ በላይ መልስ ሊኖረው ይችላል) | | | 1. የጡት እብጠት 2. የጡት መጠን 3. የጡት ጫፍ መጠንና ያለተለመደ ፈሳሽ 4. የጡት ቆዳ ቀለም መለወጥ | |
| 311 | የራስን ጡት የመመርመሪያ ዘዴዎች ምንድናቸው? | | | 1. በመመልከት 2. በመዳሰስ 3. በመመልከትና በመዳሰስ 4. አላውቅም | |
| 312 | እንዴት ነው የራስዎን ጡት በራስዎ የሚመረምሩት? | | | 1. በእጅ መዳፍና መሃል ባሉት ሶስት ጣቶች 2. በማንኛውም ጣታችን በመዳሰስ 3. አላውቅም | |
| 313 | በቁዋሚነት የራስን ጡት መመርመር ጥቅሙ ምንድነው? | | | 1. ያልተለመዱ ነገሮችን ለመለየት 2. ጡት ምን እንደሚመስልና ስሜቱን ለመማር 3. የጡት ካንሰርን ለመለየትና ለመታከም | |
| 314 | እርሶ ቱትን በራስ ለመመርመር ለታካሚዎች ወይም ለጉዋደኞችዎ አስተምረው ያውቃሉ? | | | 1. አዎ 2. የለም | |
| **3.2. ከአመለካከት ጋር የተያያዙ ጥያቄዎች** | | | | | |
| 315 | የጡት ካንሰር በከፍተኛ የሚከሰትና በኢትዮጵያ ከሁሉም ካንሰሮች የበለጠ ለሞት ምክነያት ነው፡፡ | | 1.በጣም አልስማማም  2. አልስማማም  3.አልወሰንኩም  4.እስማማለሁ  5. በጣም አስማማለሁ | | |
| 316 | እርስዎን ጨምሮ ማንኛውም ሴት በጡት ካንሰር ልትያዝ ትችላለች | | 1. በጣም አልስማማም  2. አልስማማም  3. አልወሰንኩም  4.እስማማለሁ  5. በጣም አስማማለሁ | | |
| 317 | የጡት ካንሰር የሚተላለፍ በሽታ አይደለም | | 1. በጣም አልስማማም  2. አልስማማም  3. አልወሰንኩም  4.እስማማለሁ  5. በጣም አስማማለሁ | | |
| 318 | የራስን ጡት በራስ መመርመር ካንሰርን ይከላከላል | | 1. በጣም አልስማማም  2. አልስማማም  3. አልወሰንኩም  4.እስማማለሁ  5. በጣም አስማማለሁ | | |
| 319 | የራስን ጡት በራስ መመርመር ጉዳት የለውም | | 1. በጣም አልስማማም  2. አልስማማም  3. አልወሰንኩም  4.እስማማለሁ  5. በጣም አስማማለሁ | | |
| 320 | የራስን ጡት በራስ መመርመር የማይመች አይደለም | | 1. በጣም አልስማማም  2. አልስማማም  3. አልወሰንኩም  4.እስማማለሁ  5. በጣም አስማማለሁ | | |
| - 1. **በአግባቡ ጡትን በራስ መመርመር ጋር የተያያዙ ጥያቄዎች (21-30)** | | | | | |
| 321 | እርስዎ ጡትዎን በራስዎ እንዴት እንደሚመረምሩ ያውቃሉ | | 1. በጣም አልስማማም  2. አልስማማም  3. አልወሰንኩም  4.እስማማለሁ  5. በጣም አስማማለሁ | | |
| 322 | እርስዎ የራስዎን ጡት በትክክሉ ይመረምራሉ | | 1. በጣም አልስማማም  2. አልስማማም  3..አልወሰንኩም  4.እስማማለሁ  5. በጣም አስማማለሁ | | |
| 323 | እርስዎ የራስዎን ጡት በራስዎ ምርመራ በማድረግ የጡት እብጠትን ይለያሉ | | 1. በጣም አልስማማም  2. አልስማማም  3. አልወሰንኩም  4.እስማማለሁ  5. በጣም አስማማለሁ | | |
| 324 | እርስዎ ዋኖት ፍሬ መጠን የሚያህል የጡት እብጠትን መለየት ይችላሉ | | 1. በጣም አልስማማም  2. አልስማማም  3. አልወሰንኩም  4.እስማማለሁ  5. በጣም አስማማለሁ | | |
| 325 | እርስዎ የኬቸር ፍሬ መጠን የሚያህል የጡት እብጠትን መለየት ይችላሉ | | 1. በጣም አልስማማም  2. አልስማማም  3. አልወሰንኩም  4.እስማማለሁ  5. በጣም አስማማለሁ | | |
| 326 | እርስዎ የአተር ፍሬ መጠን የሚያህል የጡት እብጠትን መለየት ይችላች | | 1. በጣም አልስማማም  2. አልስማማም  3. አልወሰንኩም  4.እስማማለሁ  5. በጣም አስማማለሁ | | |
| 327 | እርስዎ የጡት ምርመራን በራስዎ ለመመርመር ቅደም ተከተልን መሰረት አድርገው እንደሚተገብሩ እርግጠኛ ኖት | | 1. በጣም አልስማማም  2. አልስማማም  3. አልወሰንኩም  4.እስማማለሁ  5. በጣም አስማማለሁ | | |
| 328 | እርስዎ የራስዎን ጡት ሲመረምሩ ያለተለመደ ነገር ቢያገኙ መናገር ይችላሉ | | 1. በጣም አልስማማም  2. አልስማማም  3. አልወሰንኩም  4.እስማማለሁ  5. በጣም አስማማለሁ | | |
| 329 | እርስዎ የራስዎን ጡት በመስተዋት ተመልክተው ያልተለመደ ነገር ሲያዩ መናገር ይችላሉ | | 1. በጣም አልስማማም  2. አልስማማም  3. አልወሰንኩም  4.እስማማለሁ  5. በጣም አስማማለሁ | | |
| 330 | እርስዎ ጡትዎን ሲመረምሩ ትክክለኛ የጣቶን ክፍል ይጠቀማሉ | | 1. በጣም አልስማማም  2. አልስማማም  3. አልወሰንኩም  4.እስማማለሁ  5. በጣም አስማማለሁ | | |
| **ክፍል 3.4: የራስ ጡትን በራስ ለመመርመር እንቅፋት ከሚሆኑ ነገሮች ጋር የተያያዙ መጠይቆች** | | | | | |
| 331 | የራስ ጡትን በራስ መመርመር አሳፋሪ ነው | | 1. በጣም አልስማማም  2. አልስማማም  3. አልወሰንኩም  4.እስማማለሁ  5. በጣም አስማማለሁ | | |
| 332 | የራስ ጡትን በራስ መመርመር ብዙ ጊዜ ይወስዳል | | 1. በጣም አልስማማም  2. አልስማማም  3. አልወሰንኩም  4.እስማማለሁ  5. በጣም አስማማለሁ | | |
| 333 | የራስ ጡትን በራስ ለመመርመር ማስታወሱ በጣም አስቸጋሪ ነው | | 1. በጣም አልስማማም  2. አልስማማም  3. አልወሰንኩም  4.እስማማለሁ  5. በጣም አስማማለሁ | | |
| 334 | የራስ ጡትን በራስ ለመመርመር ብቻዬን ለመሆን ቦታ አላገኝም | | 1. በጣም አልስማማም  2. አልስማማም  3. አልወሰንኩም  4.እስማማለሁ  5. በጣም አስማማለሁ | | |
| 335 | በጤና ሙያተኛ የጡት ምርመራ ከተደረገ የራስ ጡትን በራስ መመርመር አያስፈልግም | | 1. በጣም አልስማማም  2. አልስማማም  3. አልወሰንኩም  4.እስማማለሁ  5. በጣም አስማማለሁ | | |
| 336 | በማሞግራፍ የጡት ምርመራ ከተደረገ የራስ ጡትን በራስ መመርመር አያስፈልግም | | 1. በጣም አልስማማም  2. አልስማማም  3. አልወሰንኩም  4.እስማማለሁ  5. በጣም አስማማለሁ | | |
| 337 | የራሴን ጡት በራሴ መርምሬ ለመጨረስ ጡቴ በጣም ትልቅ ነው | | 1. በጣም አልስማማም  2. አልስማማም  3. አልወሰንኩም  4.እስማማለሁ  5. በጣም አስማማለሁ | | |
| 338 | የራስ ጡትን በራስ ከመመርመር ይልቅ ሌሎች የባሱ ችግሮች አሉብኝ | | 1. በጣም አልስማማም  2. አልስማማም  3. አልወሰንኩም  4.እስማማለሁ  5. በጣም አስማማለሁ | | |
| **ክፍል 3.5: የራስ ጡትን በራስ መመርመር ጠቅም ጋር የተያያዙ መጠይቆች** | | | | | |
| 339 | እኔ የራሴን ጡት በራሴ ስመረምር የርሴን ጤና እየጠበኩ ነው | | 1. በጣም አልስማማም  2. አልስማማም  3. አልወሰንኩም  4.እስማማለሁ  5. በጣም አስማማለሁ | | |
| 340 | በየወሩ የራሴን ጡት በራሴ መርምሬ በማጠናቀቄ የጡት እብጠትን በቶሎ ለማግኘት ያግዛል | | 1. በጣም አልስማማም  2. አልስማማም  3. አልወሰንኩም  4.እስማማለሁ  5. በጣም አስማማለሁ | | |
| 341 | በመደበኛ የራስ ጡትን በራስ መመርመር የጡት ካንሰር ሞትን ይቀንሳል | | 1. በጣም አልስማማም  2. አልስማማም  3. አልወሰንኩም  4.እስማማለሁ  5. በጣም አስማማለሁ | | |
| 342 | የጡት እብጠትን በራስ ጡት ምርመራ በቶሎ ከተገኘ የጡት ካንሰር ህክምና ውጤቱ ጥሩ ይሆናል | | 1. በጣም አልስማማም  2. አልስማማም  3. አልወሰንኩም  4.እስማማለሁ  5. በጣም አስማማለሁ | | |
